# Versatile, sensitive liquid chromatography mass spectrometry – Implementation of 10 μm OT columns suitable for small molecules, peptides and proteins

**DOI:** 10.1038/srep37507

**Published:** 2016-11-29

**Authors:** T. Vehus, H. Roberg-Larsen, J. Waaler, S. Aslaksen, S. Krauss, S. R. Wilson, E. Lundanes

**Affiliations:** 1 Department of Chemistry, University of Oslo, Post Box 1033 Blindern, NO-0315 Oslo, Norway; 2 Department of Engineering Sciences, University of Agder, Jon Lilletunsvei 9, NO-4891 Grimstad, Norway; 3 Unit for Cell Signaling, SFI-CAST Biomedical Innovation Center, Oslo University Hospital, Rikshospitalet, NO-0027 Oslo, Norway.

## Abstract

We have designed a versatile and sensitive liquid chromatographic (LC) system, featuring a monolithic trap column and a very narrow (10 μm ID) fused silica open tubular liquid chromatography (OTLC) separation column functionalized with C_18_-groups, for separating a wide range of molecules (from small metabolites to intact proteins). Compared to today’s capillary/nanoLC approaches, our system provides significantly enhanced sensitivity (up to several orders) with matching or improved separation efficiency, and highly repeatable chromatographic performance. The chemical properties of the trap column and the analytical column were fine-tuned to obtain practical sample loading capacities (above 2 μg), an earlier bottleneck of OTLC. Using the OTLC system (combined with Orbitrap mass spectrometry), we could perform targeted metabolomics of sub-μg amounts of exosomes with 25 attogram detection limit of a breast cancer-related hydroxylated cholesterol. With the same set-up, sensitive bottom-up proteomics (targeted and untargeted) was possible, and high-resolving intact protein analysis. In contrast to state-of-the-art packed columns, our platform performs chromatography with very little dilution and is “fit-for-all”, well suited for comprehensive analysis of limited samples, and has potential as a tool for challenges in diagnostics.

The future of biomarker discovery/diagnostics/precision medication will likely include metabolomics and proteomics^1^,^2^ of small samples (dry blood spots, exosomes etc). Liquid chromatography- mass spectrometry (LC-MS) is firmly established as the technique of choice for small molecule-based diagnostics, and is emerging as a tool for protein-based diagnostics^3^. Although LC-MS is generally considered a sensitive approach, the inner diameters (IDs) of traditional LC columns are several millimetres, severely diluting samples prior to detection. This is a key problem moving towards small sample analysis e.g. tumor derived exosomes and circulating tumor cells, which are emerging as focuses of biomarker discovery. Downscaling the column ID improves sensitivity^4^, but can be associated with reduced sample capacity, chromatographic performance and/or poor column reproducibility. However, in contrast to particle packed LC columns, open tubular (OT)^5^,^6^,^7^,^8^ columns with very narrow inner diameters can offer high chromatographic efficiency, and are rather simple to produce^9^. OTLC columns have historically been shunned due to difficult handling, but these issues are gradually being resolved because of increasing availability of pumps that can handle low nL/min flow rates, low dead-volume connectors etc. Open tubular columns have in recent years been used for e.g. ultra-small sample analysis^9^. However, practical implementation of OTLC columns is however still obstructed by poor sample capacity, which can in practice counteract sensitivity gains.

Future precision medication will require comprehensive sensitivity challenging metabolome and proteome analyses. Top-down proteomics (including separation of intact proteins) will be invaluable for assessing e.g. post-translational modifications, but there are few options LC-wise for sensitive top-down proteomics^12^,^13^. In other words, there is room for versatile LC options in future clinical applications.

We have designed an OTLC-based system that has the same sample capacity and separation efficiency a larger particle-packed column nanoLC systems, while featuring significant improvements in sensitivity as promised by “ancient” OTLC theory^14^. The 10 μm ID separation column features octadecylsilane (ODS, i.e. C18) functional groups bound to a silica skeleton that coats the wall of the columns. In contrast to previous 10 μm OT variants (with a poly(styrene-co-divinylbenzene) = PS-DVB layer^9^), our ODS-OT column is suited for resolving small molecules, peptides and intact proteins, allowing same-system metabolomics and bottom-up/top-down proteomics. The combination of a 50 μm ID trap column (TC) and a 10 μm ID ODS-OT analytical column hyphenated to a high resolving Orbitrap MS is here called “attoLC-MS”. We here demonstrate traits and bioanalytical applications of attoLC-MS, towards its use in e.g. diagnostics.

## Results

### Preparation of the trap and ODS-OT columns and attoLC-MS system set-up

In addition to LC system downscaling, sample capacity is affected by the choice of stationary phase. In our system, a high sample capacity 50 μm × 40 mm poly(styrene-co-octadecyl-co-divinylbenzene) (PS-OD-DVB) monolithic trap column was employed (Supplementary Figure 1); introduction of octadecyl into the more common PS-DVB column provided at least two times higher sample capacity (>2000 ng on a 10 cm column), which is comparable to the capacity of commercial particle-packed columns (Supplementary Figure 2).

**Figure 1:**
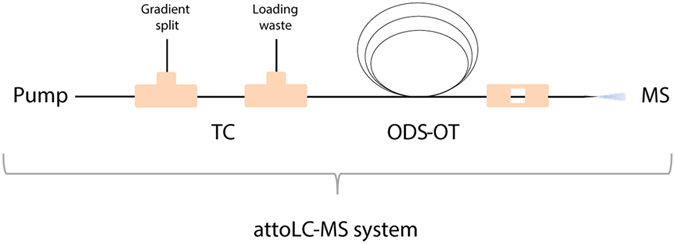
attoLC-MS system setup with trap column (TC) and ODS-OT column coupled to a mass spectrometer.

Downstream to the trap column was a 10 μm × 3000 mm OT column functionalized with octadecyl groups (Supplementary Figure 3). The procedure the column preparation was modified from a procedure for making 250 μm ID columns in capillary electrophoresis^15^. These two columns/phases matched well, providing compound refocusing due to a larger retention factor of compounds on the substantially longer separation column, allowing nanoLC-scale injections (μL/μg) on the attoLC-MS system. The columns were connected as described in ref. 9, and as shown in Fig. 1, using a commercial pump with non-modified, automated software control. The set-up of the attoLC-MS system (trap column (n = 3) + analytical column (n = 3) + connections) was highly repeatable; the total variation was less than 5% RSD for both retention time and chromatographic efficiency (Supplementary Figure 4). This is well below the required variation according to FDA guidelines^16^, and indicates that methods developed on this system will be reproducible.

#### Benchmarking the attoLC-MS system with standard LC-MS systems

**Figure 2:**
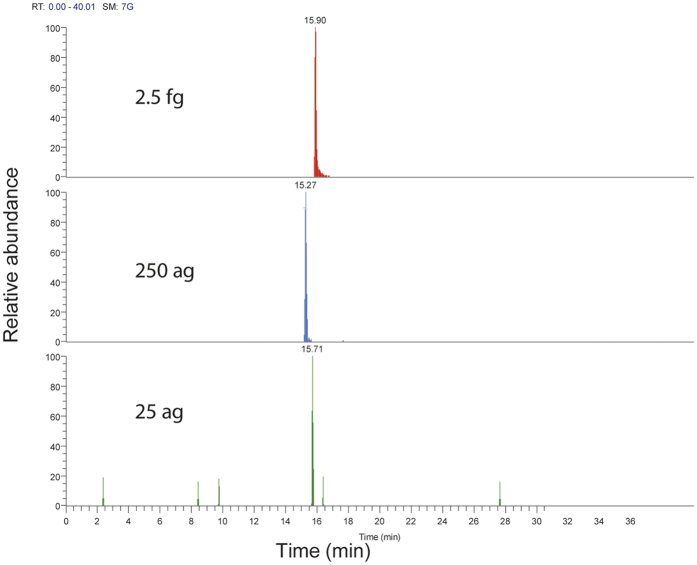
Extracted ion chromatograms of 2.5 fg, 250 ag, 25 ag of 25-d_6_ hydroxycholesterol chromatographed on the attoLC-MS system.

**Figure 3:**
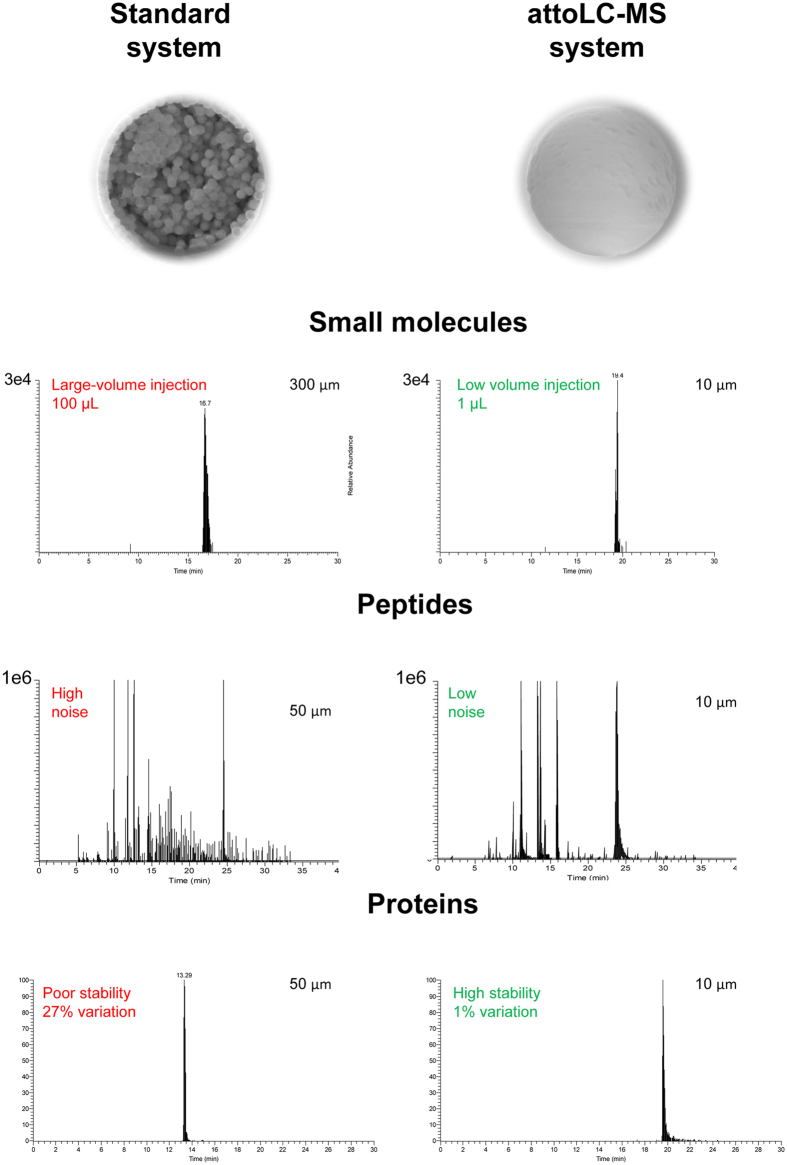
Comparison of chromatograms obtained with - standard nano LC system (i.e. particle packed columns) and ODS-OT system. LC-MS and LC-MS/MS conditions can be found in Supplementary Table 1.

Sensitivity and separation: As little as 25 ag hydroxylated cholesterol (single charged after derivatization^17^, parallel reaction monitoring, PRM) could be detected on the attoLC-MS system (Fig. 2). This is equivalent to ~50 zeptomole of analyte on the column. Similar detection capabilities have been achieved in OT format regarding peptides^10^. Compared to a standard LC-system^18^, 100 times less sample could be introduced, and still achieving the same signal (Fig. 3). Using a linear gradient on the attoLC-MS system, hydroxylated cholesterol (OHC) isomers could be baseline/satisfactory separated from each other (Supplementary Figure 5). Although the attoLC columns have meter-scale length, OHC retention times are similar to standard LC format^18^ (t_R_ about 15 minutes in both cases), as the linear velocity of the attoLC mobile phase is significantly higher. Re-equilibration times (pressure-dependent when using Thermo nanoLC pumps) are also similar, as long, open columns generate approximately the same back-pressure as shorter, particle-packed columns.

Regarding tryptic digest double- and triple-charged peptides, a more modest but clearly significant improvement in sensitivity (5-fold, data dependent MS/MS) was achieved (Fig. 3), with a flow-rate reduction from 200 nL/min (50 μm ID standard column) to 10 nL/min (attoLC). In addition, the S/N-ratio was also much larger with the attoLC-MS system (Fig. 3). Tryptic peptides were equally or better resolved on the attoLC-MS system compared to the standard system, with a peak capacity (n_c_^**^)^19^ up to 250 at 10 nL/min, compared to 130 at 200 nL/min (same t_G_/t_M_, gradient time/system hold-up time) (Supplementary Figure 6A).

For intact proteins, the 50 μm ID standard system and the attoLC-MS system (see Fig. 3) appeared to perform equally in terms of peak shape and sensitivity. However, the standard system had a very poor retention time repeatability (27% RSD), which may be explained by poor protein diffusion repeatability in the particles^20^. In contrast, the attoLC system provided far lower variation (1% RSD).

#### Application - Attogram detection limits, lipid separation and 27-OHC determination in exosomes

**Figure 4:**
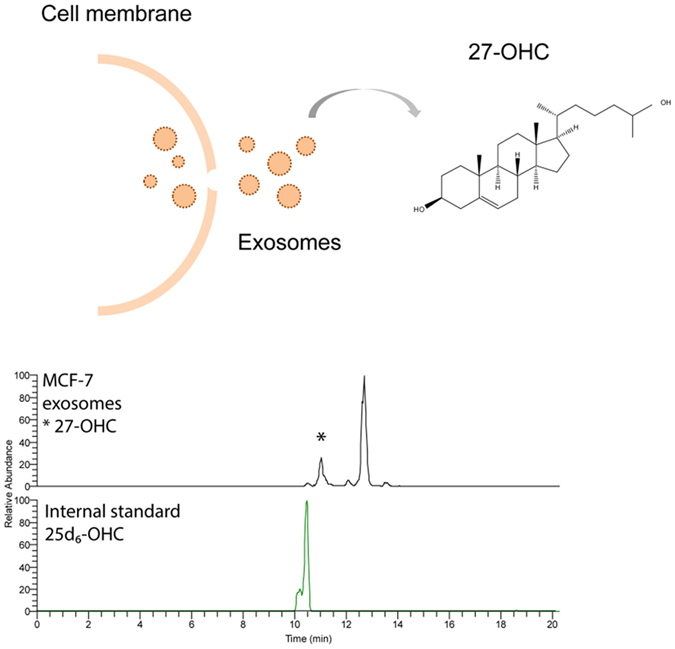
Extracted ion chromatogram of 27-OHC (*) and internal standard 25d_6_-OHC in MCF-7 exosomes chromatographed on the attoLC-MS system. LC-MS conditions are described in Supplementary Table 1.

27-OHC is significantly associated with proliferation and metastasis in estrogen positive breast cancer^21^. 27-OHC is also enriched in exosomes of ER+ cancer cell lines^18^. However, exosomes collected from patients are limited, highly valuable samples, so methods requiring low sample sizes are greatly appreciated, especially in initial biomarker-discovery stages. The attoLC-MS set-up was able to detect 27-OHC in exosomes from MCF-7 cells (Fig. 4), with just 10 ng exosomes while typical amounts for exosome lipidomics are between 10–100 μg^22^. No sample clean-up was required prior to analysis, and did not include a cholesterol-removing step, attesting to decent robustness of the system. However, column robustness investigations will be further examined, regarding e.g. temperature, clogging, pH etc.

#### Application - Detection of AXIN1 by a signature peptide in mouse embyonic stem cells (mESCs)

**Figure 5:**
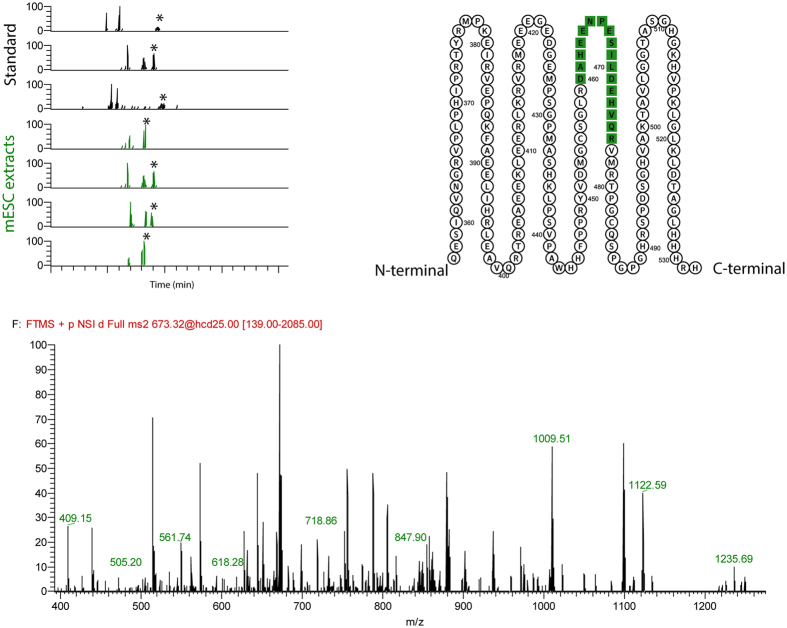
Top left: Extracted ion chromatograms of standards (n = 3) (black) and mESC extracts (n = 4) (green) with identified peptide peaks (*). Top right: partial amino acid sequence of mouse AXIN1 with identified peptide sequence (green). Bottom: MS/MS spectrum of identified peptide with annotated fragment masses in green.

The Wnt/beta-catenin-pathway is involved in many biological processes, and has been implicated as proliferating pathway in e.g. colon cancer^23^,^24^. One key protein in this pathway is AXIN1, which regulates destruction of beta-catenin (a transcriptional activator)^25^,^26^. In most biological laboratories, western blot is the go-to-technique for protein quantification. However, the success of these assays is dependent on selective antibodies^27^. Available antibodies for mouse AXIN1 has proven to be difficult to validate, due to no/poor target selectivity (i.e. no references or validation data)^28^. An alternative approach is to use LC-MS/MS based identification and quantification based on synthetic peptides or SILAC mixtures^29^,^30^. As shown for peptides (Supplementary Figure 5B), the signal increase was about 5-fold at 25 nL/min for the attoLC-MS system compared to a standard nanoLC-MS system, and thus we wanted to investigate if the atto-LC-MS system could be applicable for AXIN1 determination in mESCs through tryptic peptides, as we have previously struggled to detect this compound with LC-MS. An AXIN1 signature peptide in extracts from mESCs could indeed be successfully determined on the attoLC-MS with >3 fragment masses and within 1 minute retention time window relative to reference peptide (derived from recombinant AXIN1) (Fig. 5).

In addition to performing well for targeted proteomics identifications, comprehensive data analysis revealed that in the 5 mm gel piece between 90–100 kDa (n = 4) from which AXIN1 was extracted, 1000 proteins could be identified in 60 minutes (Supplementary Excel File).

#### Application – Intact protein separation and identification

**Figure 6:**
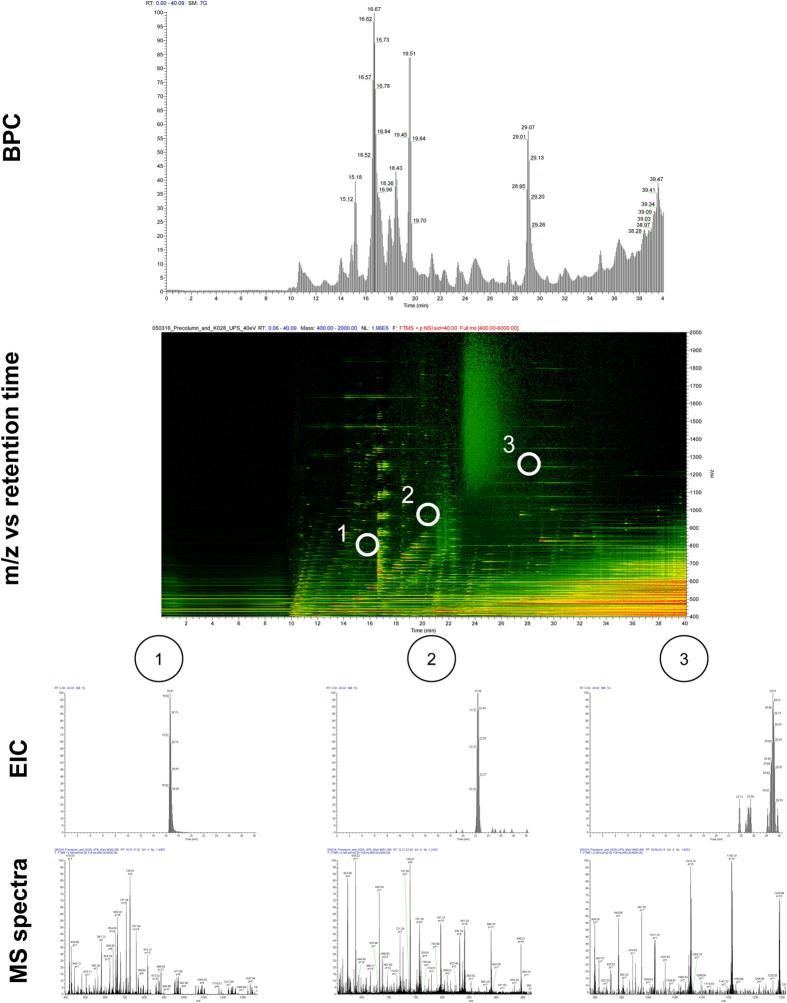
Top: Base peak chromatogram (BPC) from m/z 600–2000 of 1 μg universal protein standard (USP-1) chromatographed on the attoLC-MS system at 50 nL/min for 40 minutes with gradient composition as stated in Supplementary Table 1. Middle: Intensity of ions and *m/z* vs retention time for the sample chromatograhed; black – background.

Top-down proteomics (i.e. not performing a tryptic digestion, but analysing intact proteins directly) can provide complementary sequence/post-translational modification (PTM) information to bottom-up proteomics^13^. However, a bottleneck of this approach is that proteins are notoriously difficult to chromatograph compared to e.g. peptides and small molecules^31^. Normally, several separation techniques are combined e.g. ion-exhange chromatography, electrophoresis, isoelectric focusing^13^. LC based systems for top-down proteomics are lacking, especially regarding high sensitivity columns (i.e. nanoLC-scale or lower). The attoLC-MS system provided efficient and relatively fast protein separation and detection. The universal (USP-1) protein standard (containing 48 protein species), often used for benchmarking proteomics systems, was chromatographed satisfactory without any reduction or alkylation (Fig. 6, top). Figure 6, middle exemplifies high-resolution chromatography with three of the protein species (ubiquitin, hemoglobin and leptin) and defined MS-spectra (Fig. 6, bottom).

## Discussion

Central challenges in miniaturized separation systems include low sample capacity^19^, under-par column-to-column performance^19^ and reduced chromatographic robustness. We have developed an OTLC-MS system (attoLC-MS) with repeatable manufacturing of columns and set-up. The attoLC-MS system has high sample capacity (>2 μg) and excellent chromatographic traits. The procedure for preparing the open tubular attoLC columns was adapted from Ortiz-Villaneuva *et al*.^15^, who used larger ID column for capillary electrophoresis. Attempts to separate small molecules on PS-DVB OT columns has been provided unsatisfactory chromatography (i.e. wide peaks, large asymmetry etc.), and has hence mostly been used for peptides and proteins^32^. Further downscaling will probably increase the separation efficiency further, but will require more specialized fittings.

The attoLC column was on-line coupled with a trap column to e.g. enable μL-scale sample injections and desalting^33^. Specific attention was given to develop a trap column that could enrich relatively large sample amounts as open tubular LC systems traditionally suffer from poor sample capacity. PS-OD-DVB monolith combined with an OT column provides minimal dead-volume, excellent sample capacity and a retention factor compatibility with the ODS-OT analytical column. Other possibilities, such as silica-based monoliths^34^,^35^ may also be used, but have in our hands been difficult to manufacture repeatably.

Signal enhancements were observed with attoLC-MS, and drastically so for small, single charged molecules; attoLC-MS enabled low attogram sensitivity of hydroxycholesterols “charge tagged” with Girard T reagent^36^,^37^. This is a 100-time improvement compared to that obtained with our “regular” nanoLC-MS system^38^. Similar sensitivity were observed for other small molecules as well (e.g. sulfonamides, Supplementary Figure 7). Although significant sensitivity gains were observed for peptides as well, no sensitivity improvement was gained for intact proteins. Hence, adjusting the LC parameters will not alone ensure sensitivity enhancements for all molecules. Additional efforts to manipulate solvation effects in the electrospray process are probably needed. A larger number of water molecules surround the proteins/peptides compared to e.g the OHCs^39^,^40^,^41^, associated with a higher lower solvation energy^42^ and hence a more troublesome emission from electrospray droplets^43^.

For top-down proteomics (i.e. identification and measurements of intact proteins) a bottleneck is also the absence of suited separation columns providing high-resolution separations, stable retention times and low carry-over^13^. Hence, extensive off-line sample clean-up/fractionation prior to MS detection is undertaken^13^. Such approaches are relatively time-consuming, and loss of sample can occur. An alternative is to use a single separation and detection system, but this approach required a higher separation ability of the system (i.e. high peak capacity). For proteins, the attoLC-MS system provided a peak capacity of approximately 100 using a relatively fast 30-minute gradient, showing promise for simplifying top down proteomics. The attoLC retention time repeatability for proteins was satisfactory, and is trait promising as this feature is crucial for confident determination of compounds.

## Conclusions

We have designed a large capacity (>2 μg) nano LC system using easily prepared monolith and 10 μm OTLC columns. The system (attoLC-MS) is versatile and can be applied for metabolites, peptides and proteins in simple mixtures and more complex samples (here demonstrated with exosome extracts, mouse embryonic stem cell extracts and protein mixtures), featuring high sensitivity/resolution/repeatability.

## Experimental

### Chemicals and materials

All water used in this study was type 1 water from MilliQ (Millipore, Billerica, MA, US) unless otherwise stated. Toluene, acetone (LiChroSolv^®^), acetonitrile (ACN, HiPerSolv CHROMANORM), technical ethanol (EtOH), HCl (37 *wt*%), p-xylene, tetrahydrofuran (THF, >99.7%) methanol (MeOH, HiPerSolv CHROMANORM) and HPLC grade water were from VWR (Radnor, PA, USA). Glacial acetic acid was from Merck KGaA (Darmstadt, Germany) and absolute ethanol from Kemetyl (Vestby, Norway). 1-decanol, 1-octadecene, 1-propanol, 2-propanol, 2,2-diphenyl-1-picrylhydrazyl (DPPH), 3-(trimethoxysilyl)propyl methacrylate (γ-MAPS), 25-hydroxycholesterol (25-OHC, cholest-5-ene-3β,25-diol), 22S-hydroxycholesterol (22S-OHC, cholest-5-ene-3β,22(S)-diol), 22R-hydroxycholesterol (22R-OHC, cholest-5-ene-3β,22(R)-diol), cholesterol oxidase (from Streptomuces sp.), β-Mercaptoethanol, chlorodimethyloctadecylsilane, Dithiothrethiol (DTT), divinylbenzene (DVB), DMEM/F12+ GlutaMax, dimethylsulfoxide (DMSO), foetal bovine serum (FBS), Girard T regent, human serum albumin (HSA), lauroyl peroxide (LP), luteinizing hormone releasing hormone fragment (LH-RH fragment), insulin, iodoacetamide (IAM), N,N-dimethylformamide (DMF), NaOH, oxytocin, polyethyleneglycol (10,000, PEG), Ribonuclease A, styrene, sulfamethazine, tetramethoxysilane (TMOS), trifluoroacetic acid (TFA), urea and USP-1 protein standard were from Sigma-Aldrich (St.Louis, MI, USA). Trypsin/LysC was aquired from Promega (Fitchburg, WI, USA). Tris-HCl pH 8.0 was from Oslo University Hospital (Oslo, Norway). ESGRO were from Millipore. 20S-proteasome was from Enzo Life Sciences (Farmingdale, NY, USA). PD0325901 and CHIR99021 was from Selleck Chemicals (Houston, TX, USA). 24S-hydoxycholesterol (24S-OHC, cholest-5-ene-3β,24(S)-diol), 27-hydroxycholesterol (27-OHC, cholest-25(R)-5-ene-3β,26-diol, also known as 25^®^,26-hydroxycholesterol) (both from Avanti Polar Lipids, Alabaster, AL, USA) and 25-hydroxycholesterol -26,26,26,27,27,27-d6 (25-d6-OHC) cholest-5-ene-3β,25-diol-d6, CDN isotopes, Quebec, Canada).

C18 solid phase extraction cartridges were from Agilent (Santa Clara, CA, USA). Polyimide coated fused capillaries (360 μm outer diameter (OD), with either 10, 20 or 50 μm ID) was purchased from Polymicro Technologies (Phoenix, AZ, USA).

### Sample preparation

#### Oxysterol samples

The oxysterols were derivatized with Girard T reagent as described in ref. 38. Aliquots of 80 μL 0.402 ng/mL of each of 25-OHC, 24S-OHC, 22S-OHC, 27-OHC and 22R-OHC were mixed with 25 μL of 0.612 ng/mL 25d6-OHC and evaporated into dryness on a SC110 Savant SpeedVac (Thermo Scientific, Waltham, MA, USA) and re-dissolved in 20 μL 2-propanol (pa. Sigma-Aldrich) and added 200 μL 30 μg/mL cholesterol oxidase in 50 mM phosphate buffer pH7. This solution was incubated at 37 °C to convert the 3β-hydroxy-5-ene to a 3-oxo-4-ene. Aliquots of 500 μL of a mixture containing 15 mg Girard T regent, 15 μL glacial acetic acid and 485 μL methanol was added to the solution and the reaction was completed in the dark overnight at room temperature.

Derivatized oxysterol standard solutions were prepared as described in ref. 38. More specific, 80 μL of 1 nM 27-hydroxycholesterol standard solution was oxidized with cholesterol oxidase and reacted with Girard T reagent, to give 108 pM “MS-friendly” charge-tagged oxysterols.

Cell samples (50 000 3T3 cells) was lysed in in 100 μL absolute ethanol containing 155 ng/mL cholesterol-25-26-27-^13^C. These samples were spiked with 80 μL 0.402 ng/mL of all oxysterols and 25 μL 0.612 ng/mL 25d_6_-OHC and evaporated into dryness. Subsequently they were re-dissolved in 20 μL 2-propanol and subjected to the same derivatization procedure as described above.

#### Peptide samples

HSA tryptic digest was made by dissolving 1 mg/mL HSA protein in 50 mM Tris-HCl pH 8.0 with 8.0 M urea. DTT was added to a final concentration of 5 mM and the mixture was heated for 45 minutes at 37 °C. After cooling to room temperature, IAM was added to a concentration of 15 mM, mixed with a whirlmixer and kept in darkness for 30 minutes at room temperature. To the reduced and alkylated HSA, 20 ng trypsin/lysC were added and the solution was incubated for 4 hours at 37 °C. After 4 hours, the urea concentration was lowered below 1.5 M with addition of 50 mM Tris-HCl pH 8.0, and incubation was proceeded over night at 37 °C.

The resulting peptides were desalted using C18 extraction cartridges by the manufacturers protocol, described in detail in ref. 44.

#### Mouse embryonic stem cells (mESCs)

Mouse embryonic stem cells were cultured under standard conditions in DMEM/F12+ GlutaMax, 10^−4^ M β-Mercaptoethanol, 10% ES qualified FBS, 10^6^ U/l ESGRO, 0.5 μM PD0325901 and 3 μM CHIR99021. The cells were lyzed using a standard protocol as described in ref. 44 and protein extracts were loaded onto a 3–8% Bis-Tris Acetate gel (NuPAGE, Invitrogen, CA, USA) and separated at 150 V. The gel was subsequently stained in Coomassie Blue staining solution according to a standard protocol described in Johnsen *et al*.^45^. In-gel digestion were performed by a standard protocol supplied by the manufacturer with Trypsin/LysC. Recombinant AXIN1 was acquired from Trevor Dale, Cardiff University (UK) and sample treatment was as described above.

#### Protein samples

Ribonuclease A was dissolved in 50 mM Tris-HCl pH 8.0 to give a final concentration of 1 mg/mL. Subsequently, the protein solution was reduced with addition of 5 mM DTT and heating at 65 °C for 30 minutes, and alkylated with 15 mM IAM for 30 minutes at room temperature. The standard was diluted to a concentration of 1 ng/μL with 2% (*v/v*) ACN in 0.1% (*v/v*) TFA.

USP-1 protein standard and 20S-proteasome standard were diluted to a final protein concentration of 10 ng/μL each with 2% (*v/v*) ACN in 0.1% (*v/v*) TFA.

#### Preparation of PS-DVB and PS-OD-DVB trap columns

1 m × 50 μm fused silica capillaries were silanized using a previous described procedure^9^. After silanization, the capillaries were filled with polymerization solution using a N_2_ pressure bomb^9^. For the PS-DVB, the polymerization mixture contained 20% styrene, 20% DVB, 9% toluene, 51% 1-decanol and 1% LP (with respect to monomers). The PS-OD-DVB polymerization mixture contained 10% styrene, 10% 1-octadecene, 20% DVB, 10% DMF, 50% 1-decanol and 1% LP (with respect to monomers. All percentages are *w/w*). The difference between the batches were <1 *w*/*w*%. Before use, the 1 m prepared columns were cut into desired lengths of 10 cm and 5 cm, respectively.

#### Preparation of the ODS-OT column

A 3–4 m × 10 μm ID fused silica capillary was filled with 1 M NaOH and kept at 110 °C for at least 1 hour. It was subsequently flushed with water, 0.01 M HCl, acetone and dried with N_2_ using the pressure bomb^9^. Each wash step was carried out for at least 15 min. PEG was dissolved in 0.01 M acetic acid to a final concentration of 9 ± 1% *(w/w)*, and stirred at 4 °C for at least 30 minutes and filtered through a 0.45 μm filter. TMOS was then added to the PEG/acetic acid mixture to a final concentration of 17 ± 1% *(w/w).* The mixture was briefly sonicated and mixed with a whirlmixer before it was filled into the fused silica capillary with the N_2_ pressure bomb, plugged with GC septa at each end and heated for 48 hours at 40 °C. After 48 hours, the temperature was instantly increased to 200 °C, which was kept for 24 hours.

After cooling to room temperature, the capillary was washed with EtOH for at least 2 hours, and then dried with N_2_. Inspection of the silica layer was then performed with SEM.

Functionalization was done by flushing chlorodimethyloctadecylsilane/p-xylene 50/50 (*w/w*) through the capillary for at least 1 hour, plugging the ends and heating over night at 110 °C. After cooling to room temperature, the capillary was washed with THF, MeOH, MeOH/water 50/50 (*v/v*) and MeOH, respectively (each for at least 15 minutes). Finally the column was dried with N_2_ before SEM characterization and implementation in the attoLC-MS system.

#### Liquid chromatography-mass spectrometry systems

The column set-ups were connected to an Easy nLC1000 pump (Thermo) hyphenated with a Q-Exactive MS (Thermo) equipped with a nanospray source as described in ref. 9. Mobile phase A (MP A) was HPLC water with 0.1% (*v/v*) FA, and mobile phase B was ACN with 0.1% (*v/v*) FA. 2% (*v/v*) ACN with 0.1% (*v/v*) TFA was used as washing solvent and loading solvent. More detailed LC-MS and LC-MS/MS-settings for the applications presented can be found in Supplementary information.

#### Standard LC systems

The standard system used for peptides and proteins in this study consisted of a 75 μm ID × 2 cm trap column packed with ReproSil Gold 300 Å, 3 μm C18 particles (Dr. Maisch GmbH, Ammerbuch-Entringen, Germany) in combination with a 50 μm ID x 10 cm analytical column packed with ReproSil Gold 120 Å, 3 μm C18 particles (Dr. Maisch). The columns were slurry packed with 80% ACN and a particle concentration of 30 mg/mL with magnetic stirring. This system was operated at 200 nL/min. Gradient conditions are described in Supplementary Table 1 (Supplementary information). The standard system used for the oxysterols is described in ref. 18.

#### Trap column-octadecylsilane-open tubular system (attoLC-MS system)

The attoLC-MS system was connected as described in detail in Rogeberg *et al*.^9^, (Fig. 1) with the following exceptions: the trap column was replaced with either the prepared 50 μm × 5 cm PS-DVB or PS-OD-DVB; the analytical column was a 10 μm × 3 m ODS-OT column.

#### Scanning electron microscopy (SEM)

SEM images of the columns were acquired using a FEI Quanta 200 FEG-ESEM (FEI, Hillsboro, OR, USA). The images were taken in low vacuum mode with large field detector (LFD) and solid state detector (SSD). Digital adjustment of brightness and contrast were done within the software.

#### Data processing

All chromatograms were analyzed using Xcalibur software (v.1.4, Thermo). Peak capacities were measured manually as described in detail in ref. 44. Selection of signature peptides were done with Skyline software^46^ (v.1.7), peptideATLAS^47^ and Uniprot^48^. Comprehensive protein identification was done with the SEQUEST algorithm in Thermo Proteomics Discoverer (v.1.4) software using standard search settings as described in detail in ref. 44.

Plotting of data was done with Excel using a template from Weissgerber *et al*.^49^. In all experiments, the n > 3. MS raw data will be made available on ProteomeXchange.

## Additional Information

**How to cite this article**: Vehus, T. *et al*. Versatile, sensitive liquid chromatography mass spectrometry – Implementation of 10 µm OT columns suitable for small molecules, peptides and proteins. *Sci. Rep.* **6**, 37507; doi: 10.1038/srep37507 (2016).

**Publisher’s note:** Springer Nature remains neutral with regard to jurisdictional claims in published maps and institutional affiliations.

## Supplementary Material

Supplementary Information

Supplementary Excel File
