# Supplementary material for: Versatile, sensitive liquid chromatography mass spectrometry – Implementation of 10 μm OT columns suitable for small molecules, peptides and proteins
Source: Sci Rep. 2016 Nov 29;6:37507. doi: 10.1038/srep37507 (PMC5126632; doi:10.1038/srep37507)
Supplement: Supplementary Information [file srep37507-s1.pdf]

Supplementary information for Versatile, sensitive liquid chromatography mass spectrometry – Implementation of 10 µm OT columns suitable for small molecules, peptides and proteins

Vehus, T.[1,2]*, Roberg-Larsen, H.[1], Waaler, J.[3]., Aslaksen, S.[3], Krauss, S.[3], Wilson, S.R.[1*], Lundanes, E[1].

[1]Department of Chemistry, University of Oslo, Post Box 1033 Blindern, NO-0315 Oslo, Norway

[2]Department of Engineering Sciences, University of Agder, Jon Lilletunsvei 9, NO-4891 Grimstad, Norway

[3]Unit for Cell Signaling, SFI-CAST Biomedical Innovation Center, Oslo University Hospital, Rikshospitalet, NO-0027 Oslo.

Supplementary methods

Chemicals and reagents

Butyl methacrylate (BMA),3ethylene dimethactrylate (EDMA) and LH-RH fragment were from Sigma Aldrich.

Trap column loading capacity

Loading capacity of BMA-EDMA column (BMA, XX EDMA, XX 1-propanol,XX XX, 1 *wt* % (with respect to monomers) LP, 70°C polymerization temperature and 2-16 hours polymerization time, detailed description in Rogeberg et.al [1], PS-DVB, PS-OD-DVB and C18 particle packed columns were investigated.

Loading capacity of columns was investigated with a Easy nL1000 pump connected to a Dionex UV detector with a 11 nL flow-cell. UV absorption was monitored at 214, 230, 254 and 280 nm. 10 µL sample 200 ng/µL LH-RH fragment in 2% *(v/v)* ACN in 0.1% *(v/v)* TFA + 2 µL 2% *(v/v)* ACN in 0.1% *(v/v)* TFA was injected onto the trap column with a flow-rate of 500 nL/min. After 22 minutes (loading completed) a linear gradient from 2-50% for 10 minutes, 50-95% in 5 minutes, 95-2% in 1 minute and a hold at 2%*(v/v)* ACN in 0.1% *(v/v)* FA for 3 minutes at 200 nL/min was used to elute LH-RH from the column. The column was then equilibrated with H_2O in 0.1% *(v/v)* FA prior to the next injection.

Supplementary tables

Supplementary Table 1 – LC-MS and LC-MS/MS information used in the study.

**Small molecules**	
LC	
Injection volume (µL)	1
Loading volume (µL)	3
Flow-rate (nL/min)	100, 50, 25
Gradient (% mobile phase B, duration (min))	3-70 %, 30 minutes
ESI source	
Spray voltage	1.3kV
MS	
Mode	Full MS
Scan range (*m/z*)	50-750
Resolution (MS)	70,000
Max inject time (MS, ms)	200
AGC target (MS)	3e6
**Peptides**	
LC	
Injection volume (µL)	1
Loading volume (µL)	3
Flow-rate (nL/min)	100, 50, 25
Gradient (% mobile phase B, duration (min))	3-36 %, 30/60/120 minutes
ESI source	

Spray voltage	1.3kV
MS	
Mode	Data-dependent MS/MS, Top10
Scan range (*m/z*)	350-1850
Microscans	1
Resolution (MS)	70,000
Max inject time (MS, ms)	100
AGC target (MS)	3e6
Resolution (MS/MS)	17,500
Max inject time (MS/MS, ms)	50
AGC target (MS/MS)	1e5
Isolation width (m/z)	4,0
Normalized collision energy (NCE)	30
**Proteins**	
LC	
Injection volume (µL)	1
Loading volume (µL)	3
Flow-rate (nL/min)	100, 50
Gradient (% mobile phase B, duration (min))	3-95 %, 30 min
ESI source	
Spray voltage (kV)	1.3

In-source CID (eV)	0-80
MS	
Mode	Full MS
Scan range (*m/z*)	400-6000
Microscans	5
Resolution (MS)	140,000
Max inject time (MS, ms)	200
AGC target (MS)	3e6

Supplementary discussions and figures

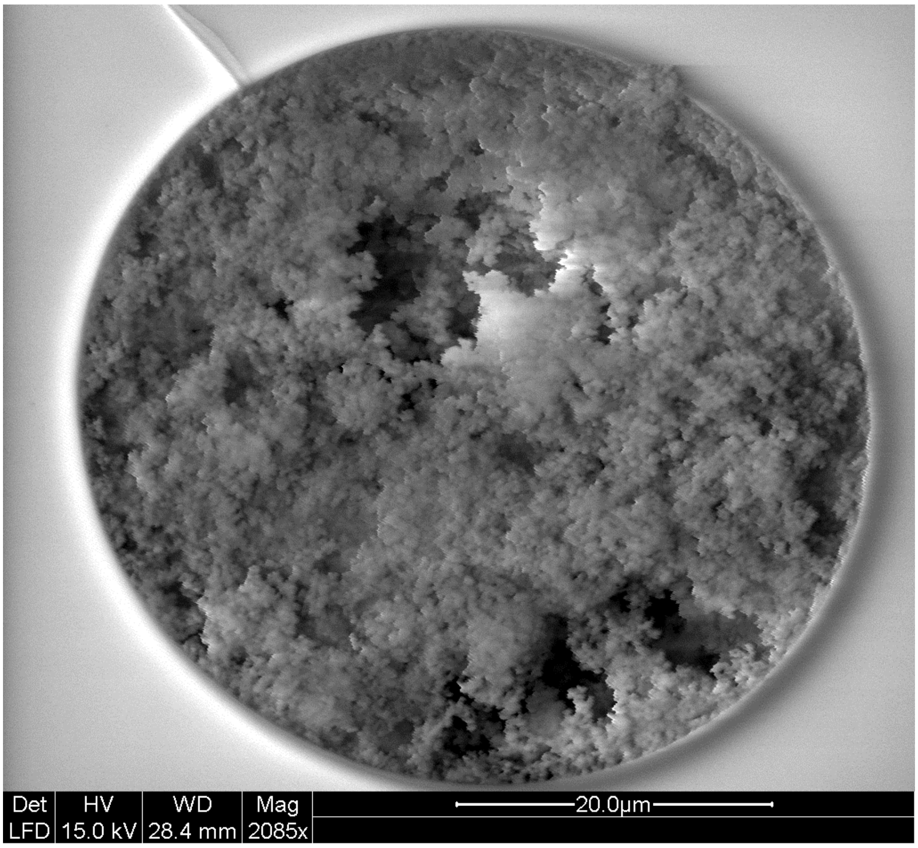

Supplementary Figure 1 – SEM image of 50 µm ID PS-OD-DVB column prepared as described in methods and materials.

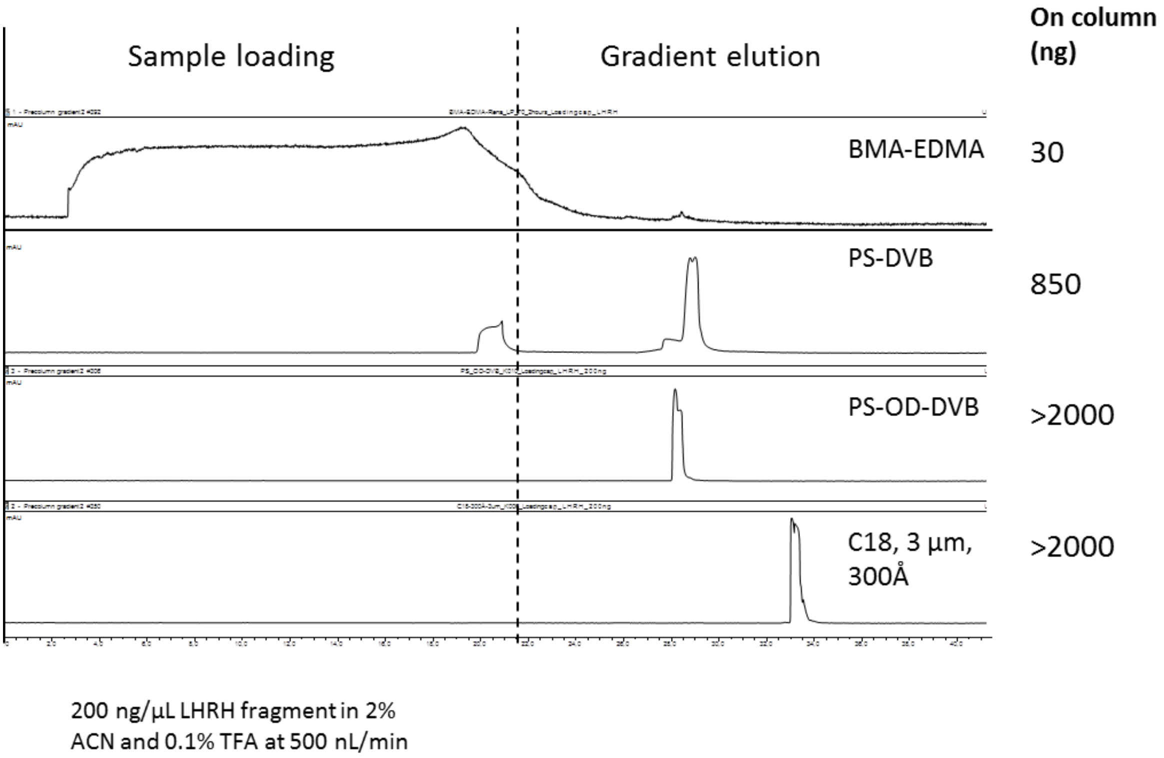

Supplementary Figure 2 – Loading capacity of 50 µm x 10 cm BMA-EDMA, 50 µm x 10 cm PS-DVB, 50 µm x 10 cm PS-OD-DVB and 75 µm x 2 cm C18 packed as decribed in methods and materials. Experimental conditions as described in supplementary methods and materials (trap column loading capacity).

Optimization of reagents used for the ODS-OT column

The ODS-OT column was made in 10 µm ID format with optimization in TMOS/PEG:acetic acid concentration and polymerization time. Optimization showed that a smooth surface without large globules was obtained with 17 ± 1 wt % TMOS in PEG/0.01 M acetic acid mixture and a reaction time of 48 hours (**Figure 1 and Supplementary Figure 3**).

A : > 25% TMOS, 24 hours at 40°C B : 17 % TMOS, 48 hours at 40°C

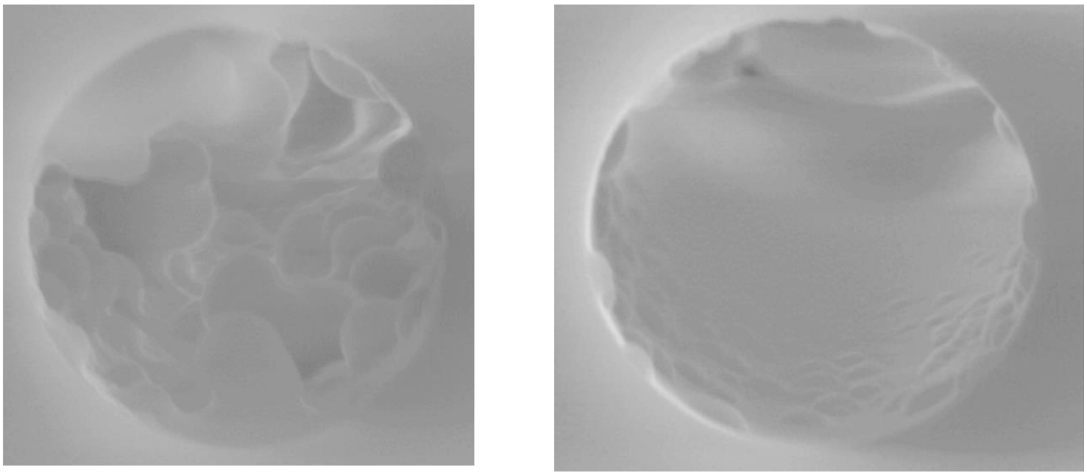

Supplementary Figure 3 – SEM images of A : ODS-OT column polymerized with more than 25 % TMOS relative to PEG:acetic acid (see Methods and Materials) and B : 17% TMOS for 48 hours (optimal conditions)

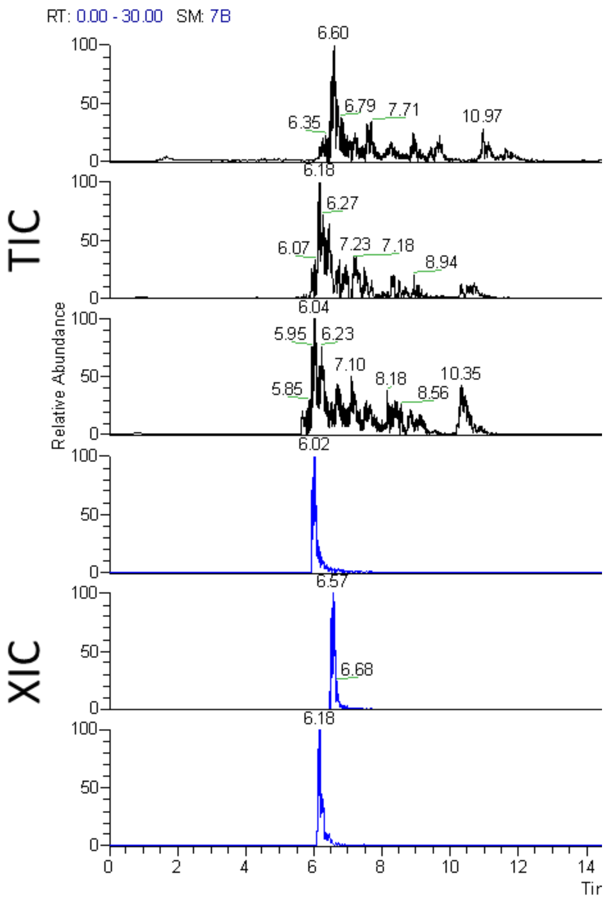

Supplementary Figure 4 – Retention time repeatability of 3 batches of TC-ODS-OT systems Upper: total ion chromatograms (TIC) Lower: extracted ion chromatograms (XIC) of 1 ng HSA tryptic digest chromatographed on the attoLC-MS system. LC-MS/MS conditions as described in Supplementary Table 1.

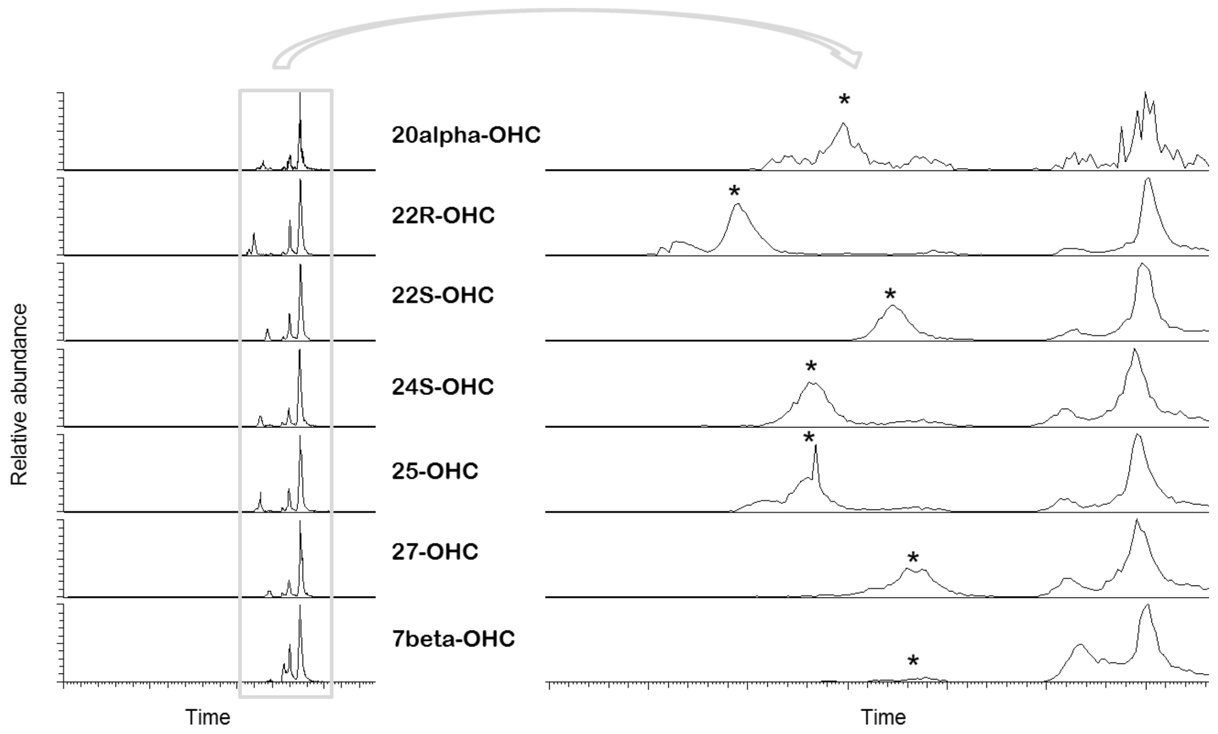

Supplementary Figure 5 - Extracted ion chromatograms showing chromatographic separation of separate injections of 20alpha-OHC, 22R-OHC, 22S-OHC, 24S-OHC, 25-OHC, 27-OHC and 7beta-OHC chromatographed on the TC-ODS-OT system at 50 nL/min and conditions as otherwise stated in Supplementary Table 1. Retention time adjusted to 25d$_6$-OHC (internal standard, not show). Asterix (*) indicates OHC peaks.

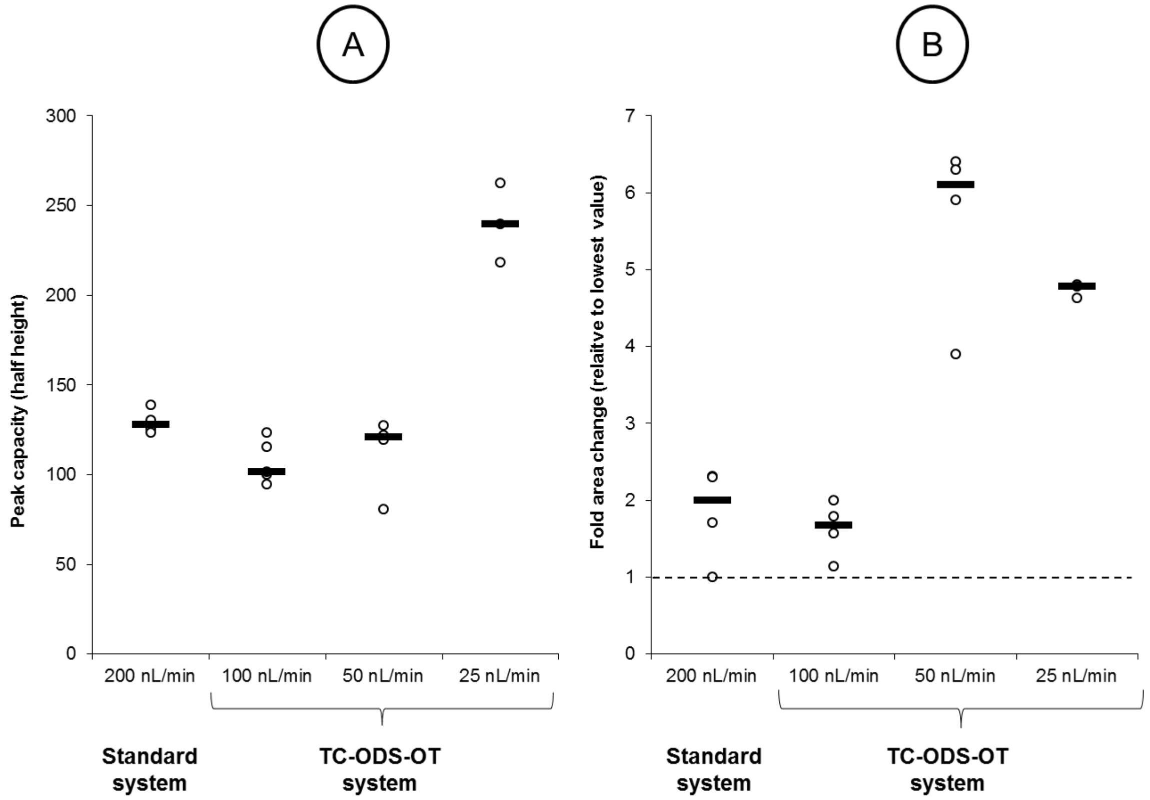

Supplementary Figure 6

A : Comparison of peak capacity of standard nano LC system with TC-ODS-OT system (at various flow-rates). Data based on chromatography of 1 ng HSA tryptic standard digest (7 peptides and 4 injection replicates per flow-rate).

B : Representative comparison of sensitivity of standard nano LC system with TC-ODS-OT system (at various flow-rates). Plot is based on single representative peptide (4 injection replicates per flow-rate).

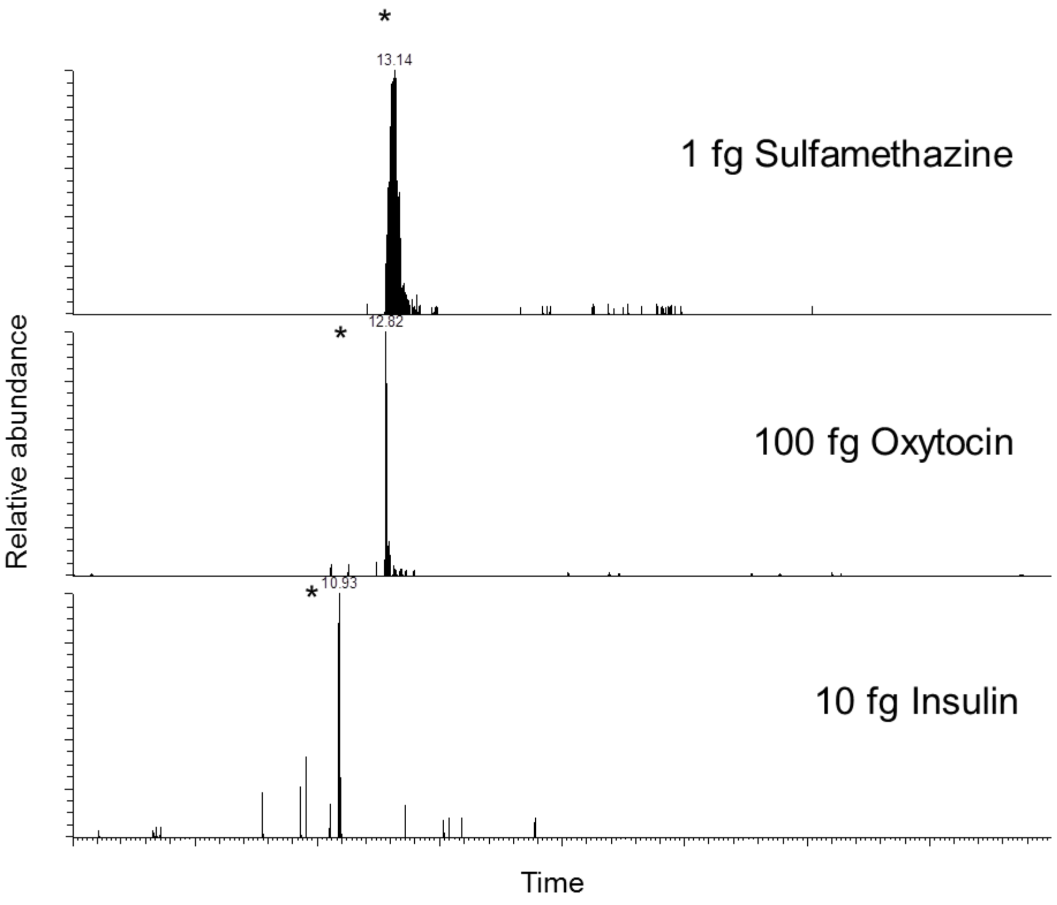

Supplementary Figure 7 – Extracted ion chromatograms of various amounts of sulfamethazine, oxytocing and insulin chromatographed with conditions as described in methods and materials.

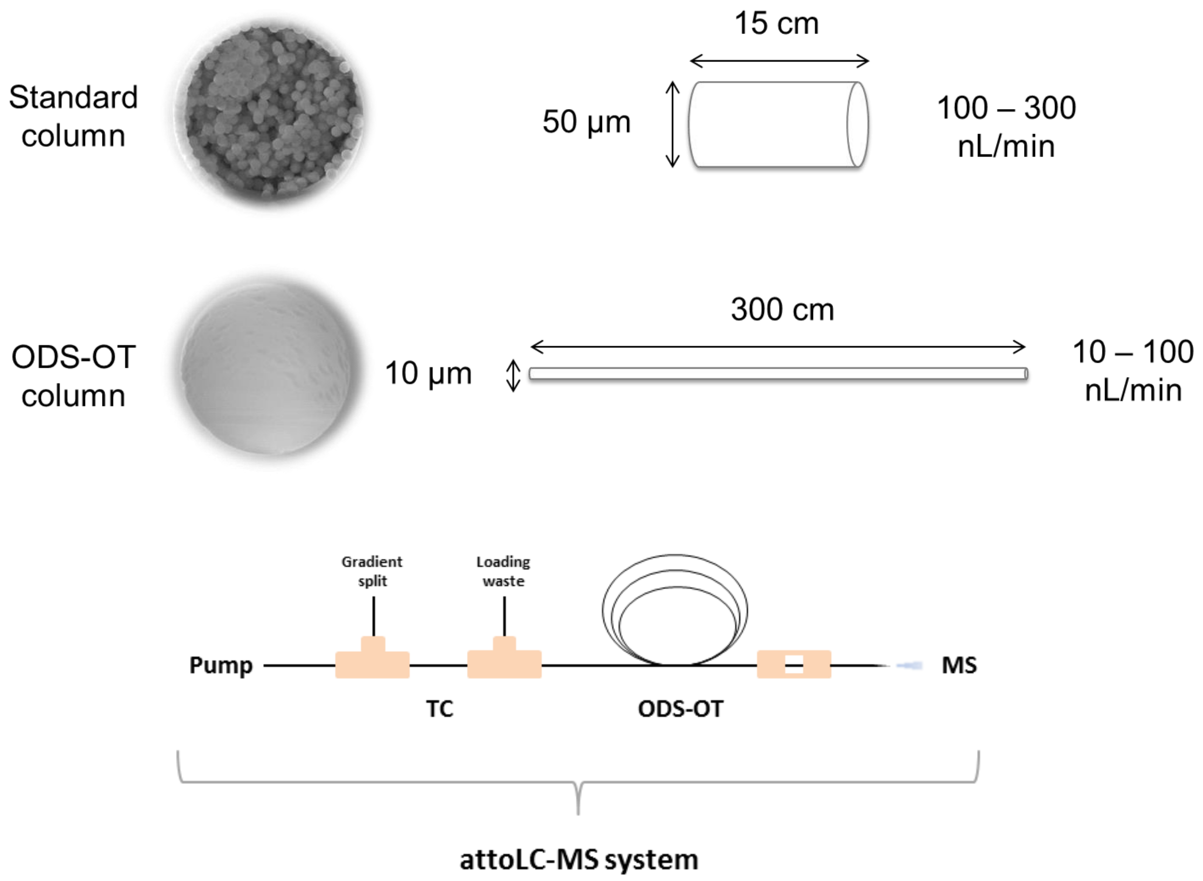

Supplementary Figure 8 – Top : Comparison of dimensions and typical flow-rates for standard columns (50 µm x 15 cm) particle packed column and atto-LC columns (10 µm x 300 cm). Bottom : attoLC-MS system setup with trap column (TC) and ODS-OT column coupled to a mass spectrometer.

Supplementary References

1. Rogeberg M, Vehus T, Grutle L, Greibrokk T, Wilson SR, Lundanes E. Separation optimization of long porous-layer open-tubular columns for nano-LC-MS of limited proteomic samples. *J. of Sep. Sci.* **36**, 2838-2847 (2013).
